# Supplementary material for: Complete symptom resolution as predictor of Helicobacter pylori eradication and factors affecting symptom resolution: Prospective follow up study
Source: PLoS One. 2021 Feb 11;16(2):e0246624. doi: 10.1371/journal.pone.0246624 (PMC7877610; doi:10.1371/journal.pone.0246624)
Supplement: S1 Table — (DOCX) [file pone.0246624.s002.docx]

**S1 Table. Predeveloped structured questionnaire (English version).**

**Part I.** **Sociodemographic data of *H. pylori* positive patients on first encounter**

| **Sr. No** | **Questions** | **Response** |
| --- | --- | --- |
| 100 | Patient Card No: ______________ | Code given_______________ |
| 101 | Sex | - Male - Female |
| 102 | Age | ______in years |
| 103 | Weight | _______Kg |
| 104 | Height | _______Meter |
| 105 | Address | Region_________________  Zone___________________  Woreda_________________  Kebele____________________  Phone No:_________________ |
| 106 | Residence | - Rural - Urban |
| 107 | Marital status | - Single - Married - Divorced - Widowed - Separated |
| 108 | Religion | - Orthodox - Protestant - Muslim - others, specify _________ |
| 109 | What is your occupation? | - House wife - Gov’t Employee - Private Employee - Merchant - Daily laborer - 7. Others, specify_______________ |
| 110 | Your educational status is? | - Unable to read and write - Read and write - Primary education(1-8^Th^ grade) - Secondary education (9-12^th^ grade) - College and above |
| 111 | Ethnicity | - Amhara - Tigrie - Agew - Oromo - Guragie - Others |
| 112 | Average monthly family income | in birr _______________ |

**Part II: Response of *H. pylori* positive patients about the disease on first encounter**

| **Sr. No** | **Questions** | **Possible responses** |
| --- | --- | --- |
| 201 | Have you ever diagnosed with H pylori infection before this? | - Yes (old) - No (New) |
| 202 | Have you taken triple therapy previously | - Yes - No |
| 203 | When your current health problem started? | - Since this week - Since last two weeks - Since a month - Since three months - Since six months - Since a year - Since two years - Before three years |
| 204 | Have you taken medications in the last two weeks? | - Yes (can you list___________) - No |
| 205 | When you feel discomfort/pain | - After meal - Before meal - Persistently or Always - At night |
| 206 | Which alcoholic drink(s) you had taken before you came for medical care? (more than one response) | - Traditional alcoholic drinks (Tella, Arekie, Teji) - Bears - Woin - Wuski - Others, specify _____________ |
| 207 | Do you have history of other chronic illnesses? (more than one response is possible) | - Liver disease - Kidney disease - Diabetes - Hypertension - Asthma - Oher_____________ |
| 208 | Do you smoke? | - Yes - No |

**Part III: Response of *H. pylori* positive patients on second encounter**

| **Sr. No** | **Questions** | **Possible responses** |
| --- | --- | --- |
| 300 | Can you give me your appointment card? | Card No: **_______________** |
| 301 | Could you stand on the balance here? | Patient weight:____________ |
| 302 | How much confident are you on your medication administration | - Surely complete (100)% - Mostly (80%) - Partially - Somewhat impossible |
| 303 | What are the major adverse drug effect(s) you encounter during therapy | _________________________________________________________________________________________________________________________________________________ |
| 304 | Have you used homemade remedies during therapy | - Yes - No |
| 305 | What modern medication other than the three drugs you took for the illness before you came here? | ___________________________________________________________________________________ |
| 306 | What you are feeling on your previous health problem now | - I am feeling nothing - Improved but still feeling - Not improved at all |

**ቀድሞ የተደራጄ መጠይቅ (የአማረኛ ቅጅ)**

**ክፍል አንድ. የጨጓራ ባክቴሪ መኖሩ የታወቀላቸው የጥናቱ ተሳታፊዎች የኢኮኖሚያዊ እና ማህበረዊ መጠይቆች**

| **ተ.ቁ** | **ጥያቄ** | **መልስ** |
| --- | --- | --- |
| 100 | ካርድ ቁጥር: ______________ | የተሰጠው ልዩ መለያ፡ _____________ |
| 101 | ፆታ፡ | - ወንድ - ሴት |
| 102 | እድሜ | ______በአመት |
| 103 | ክብደት | _______በኪሎ ግራም |
| 104 | ቁመት | _______በሜትር |
| 105 | አድራሻ | ክልል_________________  ዞን___________________  ወረዳ_________________  ቀበሌ____________________  ስልክ ቁጥር:_________________ |
| 106 | የመኖሪያ ቦታ | - ገጠር - ከተማ |
| 107 | የገብቻ ሁኔታ | - ያላገባ - ያገባ - የተፋታ - በሞት የተለያዩ - በቦታ የተለያዩ |
| 108 | የሚከተሉት ሀይማኖት | - ኦርቶዶክስ ተዋህዶ - ፕሮቴስታንት - እስልምና - ሌላ ይጠቀስ _________ |
| 109 | ስራ | - የቤት እመቤት - የመንግስት ተቀጣሪ - የግል ተቀጣሪ - ነጋዴ - የጉልበት ሰራተኛ - ሌላ ይጠቀስ፡_______________ |
| 110 | የትምህርት ደረጃ | - ያልተማረ - ማንበብ እና መጻፍ የሚችል - አንደኛ ደረጃ (1-8ኛ ክፍል) - ሁለተኛ ደረጃ (9-12ኛ ክፍል) - ከፍተኛ ደረጃ እና በላይ |
| 111 | ብሄር | - አማራ - ትግሬ - አገው - ኦሮሞ - ጉራጌ - ሌላ የገለጥ፡--------------------- |
| 112 | ወርሀዊ ገቢ | በብር _______________ |

**ክፍል ሁለት፡ የጨጓራ ባክቴሪ መኖሩ የታወቀላቸው የጥናቱ ተሳታፊዎች ስለህመማቸው የቀረበ መጠይቆች**

| **ተ.ቁ** | **ጥያቄ** | **መልስ** |
| --- | --- | --- |
| 201 | ካሁን በፊት የጨጓራ ባክቴሪያ ስለመያዝህ በምርመራ አረጋግጠህ ታውቃለህ? | - አዎ (ተገኝቶብኛል) - የለም (መጀመሪያዬ ነው) |
| 202 | የጨጓራ ባክቴሪያ የሚያጠፋ ህፍምና ወስደሀል? | - አዎ - የለም |
| 203 | አሁን እያመመህ ያለው ህመም መቼ ጀመረህ? | - ከሳምንት ወዲህ - ሁለት ሳምናት ሆኖአል - አንድ ወር ሁኖአል - ሶስት ወር - ስድስት ወር - አንድ አመት - ሁለት አመት - ከሁለት አመት በላይ |
| 204 | ባለፉት ሁለት ሳምንታት የወሰድኸው መደሀኒት አለ? | - አዎ (ምን___________) - የለም |
| 205 | የህመም ስሜቱ ሚሰማህ መቼ ነው? | - ከምግብ በኋላ - ከምግብ በፊት - ቀኑን በሙሉ - ለሊት |
| 206 | ካመት ወዲህ የጠጣሀቸውን አልኮል መጠጦች ብታሳውቀኝ? (ከአንድ በላይ መልስ ይቻላል) | - እቤት ሚዘጋጁትን (ጠላ፤ አረቄ፤ ጠጂ) - ቢራ - ወይን - ውስኪ - ሌላ ይጠቀስ _____________ |
| 207 | ከጨጓራ ሌላ ምን ህመም አለብህ? (ከአንድ በላይ መልስ ይቻላል) | - የጉበት ህመም - የኩላሊት ህመም - የስኳር ህመም - የደም ግፊት - አስም - ሌላ ይጠቀስ_____________ |
| 208 | ታጨሳለህ? | - አዎ - የለም |

**ክፍል ሶስት፡ የጨጓራ ባክቴሪ መኖሩ የታውቆ ክህምና ጨርሰው ለተመለሱ የጥናቱ ተሳታፊዎች የቀረበ መጠይቅ**

| **ተ.ቁ** | **ጥያቄ** | **መልስ** |
| --- | --- | --- |
| 300 | የቀጠሮ ካርድህን ልትሰጠኝ ትችላለህ? | ትክክለኛነቱን እና ጊዜውን ማረጋገጥ: |
| 301 | እባክህ ከክብደት መለኪያው ቁም? | ክብደት በኪሎ ግራም:____________ |
| 302 | ለህክምና የተሰጡህን መድሀኒቶች ለመውስድህ ምን ያህል እርግጠኛ ነህ? | - መቶ በመቶ - በብዛት ($\geq$80%) - በከፊል - በትንሹ |
| 303 | መድሀኒቶችን ስትወስድ ያጋጠሙህ የጎንዮሽ ጉዳቶች ምን ምን ነበሩ? | _________________________________________________________________________________________________________________________________________________ |
| 304 | ለጨጓራ ህመሙ ባህላዊ ህክምና በተላባ ወይም በአብሽ ታደርግ ነበር? | - አዎ - የለም |
| 305 | ስለጨጓራ ህመምህ ከህክምናው በኋላ ምን ይስማሀል? | - ተሽሎኛል ምንም አይሰማኝም - ቢሻለኝም የህመም ስሜቱ አለ - ምንም አልተሻለኝም |
