# Supplementary material for: Complete symptom resolution as predictor of Helicobacter pylori eradication and factors affecting symptom resolution: Prospective follow up study
Source: PLoS One. 2021 Feb 11;16(2):e0246624. doi: 10.1371/journal.pone.0246624 (PMC7877610; doi:10.1371/journal.pone.0246624)
Supplement: S2 Table — (DOCX) [file pone.0246624.s003.docx]

| **S.**  **No** | **Date of 1^st^ test** | **Name** | **Card No** | **SAT test before Rx** | **Regimen of triple therapy**  **(10 days or 14 days)** | **Date of 2^nd^ test** | **SAT test**  **after Rx** |
| --- | --- | --- | --- | --- | --- | --- | --- |
|  |  |  |  |  |  |  |  |
|  |  |  |  |  |  |  |  |
|  |  |  |  |  |  |  |  |
|  |  |  |  |  |  |  |  |
|  |  |  |  |  |  |  |  |
|  |  |  |  |  |  |  |  |
|  |  |  |  |  |  |  |  |
|  |  |  |  |  |  |  |  |
|  |  |  |  |  |  |  |  |
|  |  |  |  |  |  |  |  |
|  |  |  |  |  |  |  |  |
|  |  |  |  |  |  |  |  |
|  |  |  |  |  |  |  |  |
|  |  |  |  |  |  |  |  |
|  |  |  |  |  |  |  |  |
|  |  |  |  |  |  |  |  |
|  |  |  |  |  |  |  |  |
|  |  |  |  |  |  |  |  |
|  |  |  |  |  |  |  |  |
|  |  |  |  |  |  |  |  |

**S2 Table. *H. pylori* stool antigen test (SAT) data collection sample form**
